# Molecular Imaging Markers to Track Huntington’s Disease Pathology

**DOI:** 10.3389/fneur.2017.00011

**Published:** 2017-01-30

**Authors:** Heather Wilson, Rosa De Micco, Flavia Niccolini, Marios Politis

**Affiliations:** ^1^Neurodegeneration Imaging Group, Department of Basic and Clinical Neuroscience, King’s College London, London, UK

**Keywords:** Huntington’s disease, premanifest Huntington’s disease gene carriers, positron emission tomography, biomarkers, disease progression, pathophysiology

## Abstract

Huntington’s disease (HD) is a progressive, monogenic dominant neurodegenerative disorder caused by repeat expansion mutation in the huntingtin gene. The accumulation of mutant huntingtin protein, forming intranuclear inclusions, subsequently leads to degeneration of medium spiny neurons in the striatum and cortical areas. Genetic testing can identify HD gene carriers before individuals develop overt cognitive, psychiatric, and chorea symptoms. Thus, HD gene carriers can be studied in premanifest stages to understand and track the evolution of HD pathology. While advances have been made, the precise pathophysiological mechanisms underlying HD are unclear. Magnetic resonance imaging (MRI) and positron emission tomography (PET) have been employed to understand HD pathology in presymptomatic and symptomatic disease stages. PET imaging uses radioactive tracers to detect specific changes, at a molecular level, which could be used as markers of HD progression and to monitor response to therapeutic treatments for HD gene expansion carriers (HDGECs). This review focuses on available PET techniques, employed in cross-sectional and longitudinal human studies, as biomarkers for HD, and highlights future potential PET targets. PET studies have assessed changes in postsynaptic dopaminergic receptors, brain metabolism, microglial activation, and recently phosphodiesterase 10A (PDE10A) as markers to track HD progression. Alterations in PDE10A expression are the earliest biochemical change identified in HD gene carriers up to 43 years before predicted symptomatic onset. Thus, PDE10A expression could be a promising marker to track HD progression from early premanifest disease stages. Other PET targets which have been less well investigated as biomarkers include cannabinoid, adenosine, and GABA receptors. Future longitudinal studies are required to fully validate these PET biomarkers for use to track disease progression from far-onset premanifest to manifest HD stages. PET imaging is a crucial neuroimaging tool, with the potential to detect early changes and validate sensitivity of biomarkers for tracking HD pathology. Moreover, continued development of novel PET tracers provides exciting opportunities to investigate new molecular targets, such as histamine and serotonin receptors, to further understand the mechanisms underlying HD pathology.

## Introduction

Huntington’s disease (HD) is an inherited, neurodegenerative disorder caused by a CAG (codon that codes for amino acid glutamine) repeat expansion in the huntingtin gene (HTT) on chromosome 4 (1). The prevalence of HD varies by ethnic origin and different genetic profiles; in Caucasian populations of North America and Western Europe is 5.70 per 100,000 whereas in Asian populations is considerably lower (0.40 per 100,000) (2). The onset of symptoms is inversely associated with the size of the CAG repeat expansion and most commonly occurs at the age of mid-40s (3). However, subclinical changes and pathological processes are thought to precede the initiation of clinical symptoms by several years (4–9). There is currently no cure or disease-modifying therapy for HD, and symptomatic treatment is limited. HD will eventually lead to death, typically 15–20 years following symptomatic onset (10).

Huntington’s disease is clinically characterized by progressive motor dysfunction, cognitive decline, and psychiatric disturbances. Motor dysfunction can be divided into involuntary movements, such as chorea, and impairment of voluntary movements including bradykinesia and rigidity. Chorea presents early in the course of the disease and is most prominent in adult-onset or late-onset HD. Bradykinesia and rigidity are more dominant in earlier-onset HD and in late stages of adult-onset HD (11, 12). Significant cognitive impairments are detectable in premanifest HD gene expansion carriers (HDGECs) 10 years or less from predicted symptomatic onset, with gradual cognitive decline over the course of the disease (9, 13).

In HD, mutant huntingtin aggregates to form intranuclear inclusions, which cause neuronal dysfunction and subsequent degeneration of GABAergic striatal medium spiny neurons (MSNs) and cortical neuronal loss (14). The mechanisms underlying rate of progression from preclinical stages to the symptomatic onset are unclear. HD is potentially a good model for development of biomarkers of direct relevance to pathogenesis, since it is caused by a single gene mutation. The full penetrance of HTT mutation in people with more than 40 CAG expansions provides a unique window of opportunity to examine the neurobiological changes as they emerge in premanifest HDGECs (before the development of overt symptoms) (15) and study the clinical course of HD through to manifest stages (after motor symptom onset) (16). The identification of easily obtainable, reliable, and robust biomarkers of HD progression is crucial for the development and evaluation of disease-modifying treatments.

Previously, magnetic resonance imaging (MRI) techniques have identified structural and functional brain changes in premanifest and manifest disease stages (17). The most prominent and consistent change in HD is progressive striatal volume loss occurring about 10 years before predicted symptom onset and progressing into manifest HD stages (6–9). Cortical volume loss and white-matter changes have also been reported in premanifest stages, 10–8 years before predicted symptom onset, which also extend to manifest stages (6, 18).

**Figure 1:**
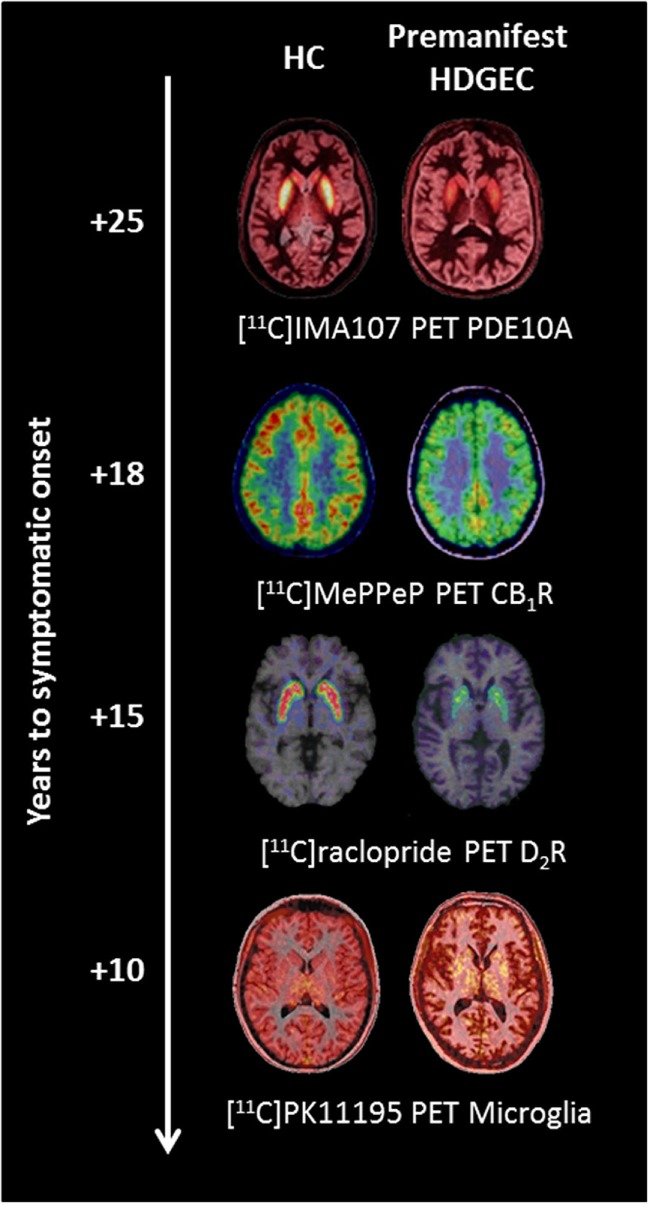
**Changes in molecular positron emission tomography (PET) biomarkers in premanifest Huntington’s disease expansion gene carriers (HDGECs) compared to healthy controls (HC) as the disease progresses toward symptomatic onset**. Axial [^11^C]IMA107, [^11^C]MePPeP, [^11^C]PK11195, and [^11^C]raclopride PET images coregistered and fused to corresponding T1-weighted MRI to show changes in premanifest HDGECs compared to HC. Earliest loss of striatal phosphodiesterase 10A (PDE10A) is detectable on average 25 years before symptomatic onset, followed by loss of cortical cannabinoid type 1 receptor (CB_1_R), increased microglia activation, and loss of striatal dopamine type 2 receptor (D_2_R) binding. Images produced from our data.

Positron emission tomography (PET) is a molecular imaging technique for the quantitative and non-invasive imaging of biological functions (19, 20). The distribution and kinetic profiles of compounds targeting specific biological molecules in tissue reflect specific biological functions in the living body. No alternatives exist for direct evaluation of human *in vivo* neurochemistry. Thus, PET molecular imaging provides a valuable tool for investigating the molecular mechanisms involved in disease progression (21). An ability to monitor the molecular markers of disease progression also has great attraction for novel drug development. In HD, PET studies have investigated changes in dopaminergic function, brain metabolism, neuroinflammation, phosphodiesterases, and cannabinoid and adenosinergic systems providing key insights into disease characteristics (17, 22). To date, alterations in phosphodiesterase 10A (PDE10A) have been identified as the earliest change occurring in premanifest HDGECs, with loss of cannabinoid receptors, loss of postsynaptic dopaminergic receptors, increased microglia activation, and increased glucose metabolism occurring as gene carriers progress toward predicted symptomatic onset (Figure 1). Therefore, PET imaging has the potential to monitor changes at a molecular level in HDGECs from early premanifest to manifest disease stages.

Here, we systematically review the current status of PET molecular imaging as a biomarker of HD pathology and disease progression and discuss the prospects for incorporating these biomarkers into future clinical trials.

## Methods

### Search Strategy

MEDLINE, ISI Web of Science, Cochrane Library, and Scopus databases electronic databases were searched for articles in English published until 25th of June 2016. Literature from abstract, conference proceedings, reviews, case-reports, and non-humans studies were not considered as a priority asset of our review. Studies were identified, combining the following major Medical Subject Headings: “Huntington’s disease” and “PET” combined with text and key words (for MEDLINE as example): ((“Huntington Disease” [MeSH Terms] OR “Huntington’s” OR “Huntington’s chorea” OR “chorea” [MeSH Terms] OR “hereditary chorea” OR “progressive chorea” OR “late onset Huntington disease” OR “juvenile Huntington disease” OR “akinetic rigid variant Huntington disease”) AND (“Positron-Emission Tomography” [MeSH Terms] OR “positron emission tomography” OR “PET”)). Additional eligible studies were identified screening the reference lists of studies included in our analysis.

### Inclusion Criteria

All selected titles and abstracts were independently reviewed by three authors (Flavia Niccolini, Heather Wilson, and Rosa de Micco). Studies were excluded if the title and/or abstract were not appropriate for the aim of the review. Full texts were subsequently obtained for eligible studies or when the relevance of an article could not be certainty excluded. Selected studies were eligible if they met the following criteria: (1) cross-sectional, case–control, or longitudinal brain PET studies including manifest or premanifest HDGECs; (2) published in peer-reviewed journals; and (3) classification as premanifest HDGECs as established by positive genetic test for CAG repeat and absence of motor signs based on the standardized total motor score (TMS) subscale (TMS = 0) of the Unified Huntington Disease Rating Scale (UHDRS) with a diagnostic confidence level of 0 (1). Reviews, case-reports, and non-human studies were excluded. PET measures included the following radioligands: [^11^C]SCH23390 (dopamine D_1_ receptors); [^11^C]raclopride (dopamine D_2_ receptors), [^11^C]FLB457 (dopamine D_2_ receptors); [^15^O]H_2_O (brain metabolism); [^18^F]FDG (glucose metabolism); [^11^C]PK11195 (microglial activation); [^11^C]IMA107 (PDE10A), [^18^F]JNJ42259152 (PDE10A), [^18^F]MNI-659 (PDE10A); [^11^C]FMZ (GABA benzodiazepine receptor), [^18^F]CPFPX [adenosine A_1_ (A_1_A) receptor], and [^18^F]MK9470 [cannabinoid type 1 (CB_1_) receptor].

## Results

A total of 44 PET studies were identified and reviewed in this article.

### Dopaminergic System

The neurodegenerative process in HD affects the two striatal projection pathways, each with distinct MSN populations. The *direct* pathway expressing mainly dopaminergic type 1 (D_1_) receptors (23) and projecting monosynaptically to the substantia nigra pars reticulata and the internal segment of the globus pallidus (23). The *indirect* pathway expressing mainly dopaminergic type 2 (D_2_) receptors (23) and projecting to the external segment of the globus pallidus (23). This leads to severe involvement of the postsynaptic dopaminergic system with relatively spared presynaptic dopaminergic nerve terminals. Therefore, although few PET studies have been performed using presynaptic dopaminergic tracers such as [^11^C]βCIT (24) and [^11^C]DTBZ (25), postsynaptic molecular imaging studies have provided further insights in the pathophysiology of motor and cognitive features in HD, showing a potential role to track HD-related pathology.

Postsynaptic dopaminergic receptors have been investigated using [^11^C]SCH22390 as a marker of D_1_ receptors, and [^11^C]raclopride and [^11^C]FLB457 as markers of D_2_ receptors. The densities of D_1_ and D_2_ receptors were significantly reduced in the striatum, ranging from 40% (24) to 75% (26) in manifest HDGECs and from 25% (27) to 50% (28) in premanifest HDGECs. Furthermore, longitudinal [^11^C]raclopride PET studies have reported annual rates of decline in D_2_ receptors from 4% (27) to 6.3% (29) and mean annual loss of D_1_ receptors of 2% (27) in premanifest HDGECs. In manifest stages, mean annual loss of D_2_ receptors of 3% and D_1_ receptors of 5% has been reported (27). MRI measures showed significant increased rates of total brain volume atrophy in premanifest and manifest HDGECs (7). A longitudinal MRI volumetric study showed premanifest HDGECs who converted to manifest HDGECs had a mean annual loss of 1.35% of white matter, 0.28% loss of gray matter, and 2.73% loss of caudate volume loss (9). Therefore, compared with mean annual rates of decline in MRI volumetric markers, loss of D_2_ receptors, as measured with [^11^C]raclopride PET, could be more sensitive to monitor disease progression. Moreover, in premanifest HDGECs, [^11^C]raclopride binding has also been shown more sensitive as disease progression biomarker than glucose metabolism in a combined [^18^F]FDG and [^11^C]raclopride longitudinal study (29).

Several studies have also found associations between decreased striatal D_1_ and D_2_ receptor levels and severity of motor impairment, as assessed by the UHDRS, suggesting that these imaging measures might be also used to stratify disease burden (27, 30). HD patients with rigidity showed more pronounced reduction of striatal D_1_ and D_2_ receptors compared to choreic HD patients without rigidity (31). In a combined [^11^C]SCH23390 and [^11^C]raclopride PET study (24), decreases in striatal D_1_ and D_2_ receptors correlated significantly with the duration of symptoms indicating that these two receptors may be reliable quantitative markers for monitoring disease progression. Decreased striatal [^11^C]raclopride binding was also associated with CAG repeat length, after correcting for age, in both premanifest and symptomatic HDGECs (28, 32). While CAG repeat length influenced the rate of striatal D_2_ receptor loss, the slopes of the correlations for premanifest were significantly different from correlation slopes for manifest HDGECs (32). This suggests that the rate of disease progression is faster during the earlier premanifest disease stages and that striatal degeneration of D_2_ receptors in HDGECs may proceed in a non-linear fashion. Thus, reduction in D_2_ receptors may be more sensitive to track disease progression in early premanifest stages than later in the disease.

Decreases in striatal D_1_ and D_2_ receptors have also been correlated with cognitive dysfunction in premanifest and manifest HDGECs (33). Similarly, decreased D_1_ and D_2_ receptor levels have been found in extra-striatal areas such as temporal (24, 34, 35) and frontal cortices (34, 35) in manifest HDGECs compared to healthy controls (HC), and correlated with cognitive impairment (24, 34, 35). Interestingly, cortical [^11^C]raclopride was decreased also in premanifest HDGECs suggesting that changes in cortical D_2_ receptor availability might be an early event in HD pathophysiology (35).

Two cross-sectional studies have investigated the extra-striatal expression of D_2_ receptors in HD (36, 37). Loss of hypothalamic D_2_ receptors in premanifest and manifest HDGECs correlated with increased microglial activation (36). In the hypothalamus, these early pathological changes could underlie the development of non-motor symptoms, including sleep disturbances, alterations in sexual behavior, and weight loss, reported in HD. Longitudinal studies are required to understand if hypothalamic D_2_ receptor dysfunction in early HD progressively deteriorates in advanced HD stages. PET with [^11^C]FLB457 investigated extra-striatal D_2_ receptors in mild to moderate HD, showing D_2_ receptors in extra-striatal regions were relatively spared; indicating D_2_ receptors outside the striatum may not be a reliable biomarker in HD (37). Progression of striatal and extra-striatal postsynaptic dopaminergic reduction has been addressed in several longitudinal studies in premanifest and manifest HDGECs. In a group of 12 manifest HDGECs (30), [^11^C]raclopride binding has been evaluated at baseline and over a 3-year period. This study showed decreased binding in striatal and extra-striatal areas, including amygdala, temporal, and frontal cortices, in HDGECs compared to HC at baseline and also revealed progressive and linear deterioration of striatal and cortical D_2_ binding overtime, although no correlation between changes in UHDRS motor scores and reductions in striatal binding was observed.

Decline in striatal and cortical dopaminergic function is detectable since the early asymptomatic stages of the disease. Dopaminergic molecular imaging might be used to predict clinical course, monitor disease progression, and track HD-related pathology, throughout the clinical features from motor to cognitive symptoms. Moreover, due to their progressive deterioration overtime, these values might also be proposed as potential biomarkers of response to novel disease-modifying and neuroprotective treatment.

### Brain Metabolism

[^18^F]FDG and [^15^O]H_2_O PET have been employed to assess regional cerebral glucose metabolism and blood flow as a marker of neuronal activity and synapses status in HDGECs. Cross-sectional [^18^F]FDG PET studies have demonstrated that striatal and cortical hypometabolism are associated with motor and cognitive symptoms in HDGECs (29, 38, 39). Decreases in caudate glucose metabolism correlated with worse bradykinesia/rigidity and total functional capacity, whereas putamen hypometabolism correlated with worse chorea, oculomotor movements, and fine motor coordination in 15 manifest HDGECs (40). Caudate hypometabolism was also associated to cognitive decline in manifest HDGECs (39, 41).

[^15^O]H_2_O PET studies have shown altered patterns of brain activation in manifest HDGECs (42–45). During motor tasks, HDGECs displayed decreased activation of the striatum, its frontal motor projection areas, and hyperactivity of the parietal areas (42), and insular regions (43, 44) suggesting that impaired striato–thalamo–cortical network may induce a compensatory recruitment of additional accessory motor pathways in HD (42–44). Moreover, during word generation tasks, manifest HDGECs showed decreased cerebral blood flows in the anterior cingulate and inferior frontal gyri and activation of the left supramarginal and right inferior frontal gyri (45). These findings suggest that HDGECs activate compensatory neuronal mechanisms in order to achieve word generation.

Striatal and cortical hypometabolism is an early feature of HD and may precede neuronal loss (46–49). Ciarmiello and colleagues (47) found that striatal and cortical hypometabolism and white-matter volume loss seen on MR imaging preceded gray-matter atrophy in 24 premanifest HDGECs. Moreover, reduced glucose levels may contribute to the time of HD onset (48, 49). Specifically, premanifest HDGECs who became symptomatic after 5 years showed significantly lower mean glucose uptake in the caudate than those who did not convert, and this difference was independent of mutation size (48). It was recently shown that also putamen hypometabolism is a predictor for symptomatic conversion in a cohort of 22 premanifest HDGECs (49). Premanifest HDGECs who phenoconverted to manifest HDGECs after 10 years had significant decreased [^18^F]FDG uptake in the putamen at 2-year follow-up compared to premanifest HDGECs who did not become symptomatic (49). A trend for worse putaminal metabolism at 2-year follow-up was observed in those premanifest HDGECs closer to symptomatic onset as compared to premanifest HDGECs far from onset (49). In early stage manifest HDGECs, glucose metabolism in frontotemporal and parietal cortices was significantly lower in those with faster progression of the disease suggesting that decreased cortical metabolism in early manifest HDGECs is indicative of rapid progression (50).

A longitudinal [^18^F]FDG and [^11^C]raclopride study showed a non-significant 2.3% mean annualized striatal hypometabolism in 10 premanifest and 8 manifest HDGECs (29). These findings suggest that [^18^F]FDG PET is a less sensitive marker of disease progression compared to [^11^C]raclopride.

[^18^F]FDG PET network analysis has been used to identify spatial covariance patterns in premanifest HDGECs (51–53). Cross-sectional analysis of metabolic changes has shown a reproducible disease-related pattern able to discriminate between premanifest HDGECs and HC (52). This disease-related pattern consisted in bilateral increases in thalamic, occipital, and cerebellar glucose metabolism associated with bilateral decreases in striatal metabolism (52). However, premanifest HDGECs did not show consistent changes over time, thus limiting the utility of this pattern as a network biomarker of preclinical disease progression. Longitudinal metabolic network analysis using changes in pattern expression in 12 premanifest HDGECs who were a mean of 10 years from symptomatic onset showed a distinct spatial covariance pattern associated with disease progression (53). Over a 7-year period, premanifest HDGECs showed a progressive decline in glucose metabolism in the caudate, putamen, thalamus, insula, posterior cingulate gyrus, prefrontal, and occipital cortices associated with associated with increases in the cerebellum, pons, hippocampus, and orbitofrontal cortex (54). Premanifest HDGECs who showed further increases in metabolic network activity at baseline (>2 SD above the normal mean) had a greater risk of symptomatic conversion in the following 5-year period (53). This metabolic progression network may provide a sensitive biomarker of disease progression in the decade prior to symptomatic onset and may be used as tool to evaluate the effect of new pharmacological therapies aiming to slow down the progression of the disease.

### Neuroinflammation and Activated Microglia

Microglial activation and altered immune responses likely play a major role in the pathogenesis of HD (55). Microglia activation can be measured *in vivo* using PET imaging with radioligands selective for 18-kDa translocator protein (TSPO), which is expressed on the surface of activated microglial (56, 57). To-date, five PET studies have used the TSPO tracer [^11^C]PK11195 to assess microglial activation in HDGECs at premanifest and manifest stages.

Positron emission tomography with [^11^C]PK11195 has revealed significant increases in microglial activation in striatal and cortical regions in premanifest (58) and manifest HDGECs (59). Increases in striatal microglial activation of 50% in manifest HD correlated with severity of striatal dopamine D_2_ receptor loss, as measured by [^11^C]raclopride PET, and with worse motor symptoms (59). In premanifest HDGECs, increased microglial activation in the associative striatum correlated with cognitive dysfunction and with higher probability of symptomatic onset over the next 5 years (36, 58, 60). Therefore, microglial activation is an early event in the course of HD and is likely involved in subclinical disease progression. Findings from PET imaging are consistent with postmortem studies, showing the presence of activated microglial in areas of neuronal loss in HD brains (61, 62). These results suggest over-activation and dysregulation of the immune response is harmful to neuronal integrity and could worsen neurodegeneration in HD.

In HD, mutant huntingtin protein is expressed throughout the body and influences dysfunction of both the central nervous system and peripheral immune cells (63, 64). Mutant huntingtin-induced immune activation, subsequently exacerbating neurotoxicity, could represent a direct link between immune activation and pathogenesis of HD (65). Increased levels of plasma pro-inflammatory cytokines, such as interleukin (IL)-6, IL-8, and tumor necrosis factor (TNF)-α, have been reported in premanifest and manifest HD, with levels increasing with advancing disease (65, 66). Furthermore, plasma levels of IL-1β, IL-6, IL-8, and TNF-α correlated with increased [^11^C]PK11195 binding in the somatosensory cortex in premanifest HDGECs who were more than a decade from predicted symptomatic onset (67). Therefore, demonstrating *in vivo* a direct link between peripheral and central immune dysfunction in HD and supporting the role of immune dysfunction in pathogenesis of HD. Furthermore, abnormal cytokine levels before symptom onset could be used a biomarker to track HD progression (68).

Increased immune response could occur in response to neuronal death induced by mutant huntingtin toxicity and thus may play a protective role in HD. However, given the harmful effects of microglial enzymes (69) and microglia driven neurotoxicity, which is exacerbated by the presence of mutant huntingtin protein (65, 70), increased microglial activation is likely to eventually exacerbate HD pathogenesis. Immune activation in premanifest HD stages could trigger subsequent striatal and cortical neurodegeneration.

Given the growing evidence for the role of microglial activation and neuroinflammation in pathophysiology of HD, it is important to further understand this process. Future PET imaging studies will likely incorporate new second generation PET tracers targeting TSPO include [^11^C]GE180 and [^11^C]PBR28, [^11^C]CB184 (71) that aims to overcome poor signal-to-noise ratio and high levels of non-specific binding associated with [^11^C]PK11195 tracer (72).

### Phosphodiesterase 10A

Phosphodiesterase 10A is a dual substrate enzyme that hydrolyzes both cyclic adenosine monophosphate (cAMP) and cyclic guanosine monophosphate, thus regulates cyclic nucleotide-mediated intracellular signaling cascades and promotes neuronal survival (73, 74). Cyclic nucleotide signaling plays an important role in signal transduction and synaptic transmission of dopaminergic, noradrenergic, serotonergic, and glutamatergic neurons (75, 76). PDE10A is most highly expressed in the striatal MSNs, where it modulates the cAMP/PKA/DARPP-32 signaling cascade, and controls the phosphorylation state and activity of many downstream physiological effectors including gene transcription factors such as cAMP-response element-binding protein, brain derived neurotrophic factor, extracellular signal-regulated kinase, and various neurotransmitter receptors and voltage-gated ion channels. Therefore, PDE10A plays an essential role in regulation of the *direct* and *indirect* striatal output pathways striatal output (77–79).

Phosphodiesterase 10A expression was first assessed *in vivo* in a small cohort of five manifest HDGECs using PET with [^18^F]JNJ42258152, which revealed signal loss of 70.7% in the caudate and 62.6% loss in the putamen (80). MRI volumetric analysis showed manifest HDGECs had significant striatal atrophy, which may affect expression of this intracellular enzyme (80). Russell and colleagues used PET with [^18^F]MNI-659 to investigate PDE10A expression in eight early manifest and three premanifest HDGECs (81, 82). Loss of 47.6% in was reported in striatal and pallidal in the combined manifest and premanifest HD cohort. The three premanifest HDGECs showed intermediate decreases in PDE10A expression compared to manifest HDGECs (81). Lower striatal [^18^F]MNI-659 binding correlated with worse UHDRS motor scores, disease burden pathology, and regional atrophy (81). Moreover, a 1-year follow-up study revealed loss of [^18^F]MNI-659 binding of 16.6% in caudate, 6.9% in putamen, and 5.8% in globus pallidus (82), thus supporting the use of PDE10A as a biomarker to track HD progression.

Recently, our group investigated PDE10A expression using PET with [^11^C]IMA107 in 12 far-onset premanifest HDGECs who were on average 25 years from predicted symptomatic onset, clinically normal and had no volumetric brain changes as confirmed by MRI voxel-based morphometry and FreeSurfer volumetric analysis (83, 84). Striatal and pallidal [^11^C]IMA107 binding was reduced by 25–33%, and a 35% increase in binding was observed in the motor thalamic nuclei in premanifest HDGECs compared to HC (83). Connectivity-based functional parcellation analysis was carried out according to cortical–striatal connectivity, and striatal connectivity with substantia nigral/globus pallidus internus (striatonigral: *direct* pathway) and globus pallidus externus (striatal–pallidal: *indirect* pathway). Prominent PDE10A decreases were confined to the sensorimotor striatum and to the striatonigral and striatopallidal projection segments (83). Furthermore, the ratio between higher PDE10A expression in motor thalamic nuclei and lower PDE10A expression in striatopallidal projecting striatum was strongly associated with higher probability of symptomatic conversion in far-onset premanifest HDGECs (83). Loss of PDE10A expression was also observed beyond the basal ganglia, specifically in the insula and occipital cortex which are affected even at the earliest stages of the disease and could play a role in the development of cognitive and behavioral symptoms in HD (84). Together, findings suggest bidirectional alterations of PDE10A signaling, within neuropathological salient networks, are an early biochemical change occurring up to 43 years before predict symptomatic onset of HD.

Recently, two genetic studies investigated PDE10A expression in patients with young onset hyperkinetic movement disorders who carry PDE10A mutations. A [^11^C]IMA107 PET study revealed a significant 70% loss of striatal PDE10A expression in an individual with biallelic PDE10A missense mutations (85). The second study identified three childhood-onset chorea patients who carried *de novo* heterozygous mutations in PDE10A (86). Substitution mutations were located in regulatory GAF-B domain, which binds to cAMP stimulating the activity of PDE10A catalytic domain. *In vitro* functional studies demonstrated substitution mutations did not affect striatal PDE10A activity but rather disrupts the stimulatory effect mediated by cAMP binding to the GAF-B domain (86). These findings confirm that PDE10A, and cAMP signaling, plays a crucial role in regulating striato-cortical movement control. Moreover, genetic depletion of PDE10A alters cyclic nucleotide signaling, resulting in over-activation of motor activity and a hyperkinetic movement disorder. The identification of PDE10A mutations as a cause of chorea further highlights the role of PDE10A in pathogenesis of HD and motivates longitudinal studies validating PDE10A as a marker to track disease progression.

Phosphodiesterase 10A is a promising biomarker of HD pathology with the potential to be used to monitor disease progression and responses to therapeutic treatments. Large scale longitudinal studies are required to confirm the relationship between PDE10A expression levels and clinical symptomatology and validate use of PDE10A and [^11^C]IMA107 as a marker to track HD pathology.

### Cannabinoid System

Cannabinoid type 1 (CB_1_) receptors are highly expressed on GABAergic striatal MSNs where they modulate synaptic transmission and thus play a key role in the pathogenesis of HD (87). CB_1_ receptor signaling modulates corticostriatal depression of glutamatergic synaptic transmission (88), and cerebellar long-term depression thus facilitating inhibition of GABAergic inhibitory postsynaptic currents (89). CB_1_ receptor messenger-RNA is one of the most consistently decreased transcripts in postmortem striatal tissue (90). CB_1_ receptor dysfunction could result in mutilation of CB_1_ receptor-dependent synaptic plasticity, subsequent loss of anti-GABAergic and anti-glutamatergic effect, loss of buffering excitotoxicity and thus, neuronal injury (91).

A PET study with [^18^F]MK-9470 demonstrated significant decreases in striatal CB_1_ receptor levels in 20 manifest HDGECs compared to HC (92). CB_1_ receptors likely play a central role in pathophysiological mechanisms underlying HD; however, it is unclear how early changes occur and their progression across disease stages. Future longitudinal studies should focus to use PET tracers specific for CB_1_ receptor expression, for example, [^11^C]MePPEP (93), to track changes in early premanifest to advanced manifest HD stages, and assess associations with disease burden scores and clinical measures.

### Adenosinergic System

Type 1 adenosine (A_1_) receptors are expressed through the human brain where they could play a neuroprotective role in pathological conditions such as excitotoxicity and inflammation (94). A PET study with [^18^F]CPFPX, a marker of A_1_ receptors, demonstrated a 25% loss of binding in the caudate of eight manifest HDGECs but no significant differences in caudate or putamen A_1_ receptor levels in premanifest HDGECs compared to HC (95). Increased thalamic A_1_ receptor levels were reported in far-onset premanifest HDGECs, but no change in thalamic receptor binding in near-onset premanifest HDGECs compared to HC (95). Furthermore, there was a strong correlation between A_1_ receptor binding and years to symptomatic onset. These findings suggest A_1_ receptors could shift from supranormal to subnormal levels during phenoconversion of HD. Differential regulation of A_1_ receptors may play a role in the pathophysiology of altered energy metabolism.

### Other PET Molecular Markers

Opioid and GABA receptors are also affected in HD (96). PET with [^11^C]diprenorphine, a marker of opioid receptors, has shown loss of 31% in the caudate and 26% loss in the putamen of five manifest HDGECs (43, 44). Loss of striatal opioid receptors is lower than striatal dopaminergic degeneration, which shows about 60% loss in manifest HD (31). Therefore, suggesting that opioid receptors are less vulnerable to disease progression and that dopaminergic degeneration is likely a stronger marker for disease progression.

Two PET studies have investigated GABA receptors in HD using [^11^C]flumazenil. In six manifest HDGECs, significant 17% loss of GABA receptors was reported in the caudate but not in the putamen or thalamus (97). Additionally, reductions in glucose uptake, measured with [^18^F]FDG, were observed in caudate, putamen, and thalamus in manifest HDGECs (97). A second PET study found similar results with reduction in GABA receptors in the caudate, but not putamen, and decreased glucose uptake in the caudate, putamen, and thalamus in HDGECs (98). Metabolic changes in the putamen and thalamus, without detectable reductions of GABA receptor levels suggest that metabolic impairment occurs before alterations in GABA receptors and thus is likely an earlier marker of disease pathology. Comparing also with [^11^C]raclopride binding, manifest HDGECs (who exhibited loss of D_2_ receptors) showed significantly less GABA receptors in caudate compared to premanifest HDGECs with normal D_2_ receptor levels, whereas premanifest HDGECs with reduced D_2_ receptors levels did not show any differences compared to manifest HDGECs (98). Therefore, suggesting loss of GABA receptors likely occurs only in manifest HD stages.

### Conclusion and Future Directions

**Figure 2:**
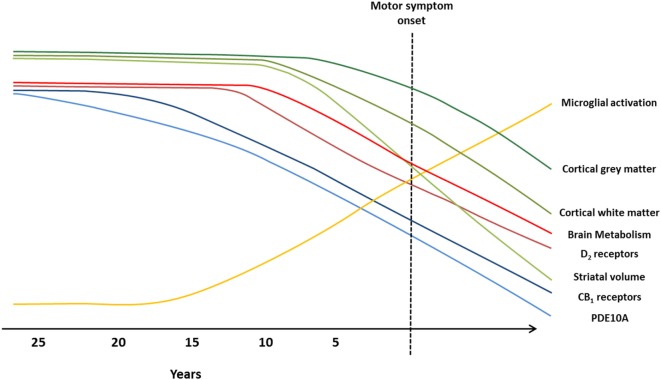
**Imaging biomarkers to track Huntington’s disease (HD) pathology from premanifest to symptomatic disease stages**. Graphical illustration of current biomarkers which have been assessed to track HD progression, from premanifest to symptomatic stages, using magnetic resonance imaging (MRI) and positron emission tomography (PET) molecular imaging techniques. Changes in markers are representative of alterations observed in MRI and PET studies, and the predicted progression over the disease course. Green lines represent MRI markers (99): loss of cortical gray matter (top, dark green), cortical white matter (middle, green), and striatal volume (bottom, light green). Blue, red, and yellow lines represent PET markers; decline in brain metabolism (top, bright red), dopamine type 2 (D_2_) receptors (bottom, dark red), cannabinoid type 1 (CB_1_) receptors (top, dark blue), and phosphodiesterase 10A (PDE10A) (bottom, light blue), and increased microglial activation (yellow).

Cross-sectional and small longitudinal PET studies have investigated the use of dopamine receptors, brain metabolism, microglial activation, and PDE10A as potential HD biomarkers. Overall, findings indicate brain metabolic changes may be a sensitive marker for disease progression in premanifest stages and could also be predictive of symptomatic conversion in HD. However, longitudinal studies revealed no changes in glucose metabolism; thus only metabolic network analysis could be useful for tracking disease progression. Moreover, glucose metabolism has been shown to be a less sensitive marker of disease progression compared to [^11^C]raclopride. To-date, alterations in PDE10A expression are the earliest biochemical change identified in HD and show promising results as a biomarker to track HD pathology. Large longitudinal studies are required to fully validate the use of PDE10A compared to other molecular markers to track disease progression in HD. Integrated PET and MRI studies directly comparing PET and MRI markers are required to fully determine which biomarkers prove to be most sensitive for monitoring HD progression. It is possible that a combination of different imaging markers could be more useful to track pathology at different disease stages (Figure 2).

There are limitations for use of the currently available biomarkers due to some inconsistent findings over longitudinal studies and specific limitation described of each ligand. While inflammation likely plays an important role in the pathophysiology of HD, microglial activation alone it is unlikely to act as reliable marker to track HD progression. There are a number of potential issues surrounding the use of immune changes as a marker to monitoring disease progression. These include the non-specificity of biomarkers, other factors contributing to increased immune responses such as other illnesses, nutritional, metabolic, and infective pathology, and variability in inflammation levels throughout the same day or over different days making reliable quantification and accurate comparisons challenging. However, the fact that mutant huntingtin is sufficient to influence abnormal activation of immune responses and changes are observed prior to symptom onset provides compelling evidence for further PET longitudinal studies to fully evaluate the specificity and sensitivity of immune changes as potential markers to track disease progression and to monitor responses to anti-inflammatory therapeutic treatments.

Future PET studies using novel tracers to investigate 5-hydroxytryptamine-2A (5-HT_2A_) receptors and histamine type-3 (H_3_) receptor function *in vivo* could provide key insights into their role in the pathophysiology in HD. Preclinical studies have suggested involvement of serotonin receptors, specifically 5-HT_2A_ receptors, in the pathophysiology of HD (100–102). Regulation of 5-HT receptors potentially has a direct neuroprotective effect on neurons and alleviating symptoms in HD. H_3_ receptors are highly expressed in the basal ganglia and cortical regions; postmortem studies in HD brains revealed lower H_3_ receptors in the caudate, putamen, and pallidum suggesting involvement of H_3_ receptors in the pathophysiology of HD (103). New PET ligands selective for 5-HT_2A_ and H_3_ receptors, which have the potential to detect longitudinal changes in receptor expression levels, will provide insights into receptor availability across premanifest and manifest disease stages aiming to understand their potential use as disease biomarkers.

## Author Contributions

HW, RM, FN, and MP all contributed to drafting of manuscript and critical revision for important intellectual content.

## Conflict of Interest Statement

The authors declare that the research was conducted in the absence of any commercial or financial relationships that could be construed as a potential conflict of interest.
